# Lupus Cerebritis as the Initial Presentation of Systemic Lupus Erythematosus in a Young Female

**DOI:** 10.7759/cureus.14259

**Published:** 2021-04-02

**Authors:** Kiranpreet Gosal, Erin Pollock, Andrew Mangano, Kevin Dao

**Affiliations:** 1 Internal Medicine, Grand Strand Medical Center, Myrtle Beach, USA

**Keywords:** systemic lupus erythematosus, lupus cerebritis, psychosis, neuropsychiatric lupus

## Abstract

The psychiatric and neurological symptoms of systemic lupus erythematosus (SLE) are referred to as lupus cerebritis. The wide range of symptoms associated with SLE can pose a diagnostic challenge. We present a case of lupus cerebritis in a 31-year-old female presenting with psychosis. We present this case to increase awareness of the psychiatric manifestations of SLE that can be mistaken for more common etiologies of psychosis.

## Introduction

Lupus cerebritis encompasses the neurological and psychiatric manifestations of systemic lupus erythematosus (SLE). These manifestations can occur quickly after or even before the diagnosis of SLE and should be considered in patients presenting with symptoms such as altered mental status (AMS), headache, anxiety, depression or unexplained psychosis. The wide range of symptoms related to lupus cerebritis pose a diagnostic challenge. Diagnosing SLE through serologic markers and biopsy, as well as ruling out other causes of nervous system dysfunction is important in establishing a diagnosis of lupus cerebritis.

This article was previously presented as a video poster at the 2020 South Carolina American College of Physicians’ Abstract Competition on September 21, 2020.

## Case presentation

A 31-year-old female presented to the emergency department with intermittent AMS and fever for one week. She had progressive mental decline during this time with visual hallucinations, which required hospital admission. She had a past medical history of genital herpes simplex diagnosed several months prior to presentation. She had no past surgical history and family history consisted of hypertension and type II diabetes mellitus. Her only recent home medication was metoclopramide, which she took for five days two months ago for nausea secondary to gastroenteritis.

Her vitals on admission were remarkable for a fever of 100.8 F, heart rate of 135 beats per minute, and blood pressure of 162/105 mmHg. On physical exam, the patient had clear lung sounds bilaterally, regular rate and rhythm, normal bowel sounds with a soft abdomen, no swelling of extremities, no motor or sensory deficits. The patient appeared agitated and was having visual hallucinations.

**Figure 1:**
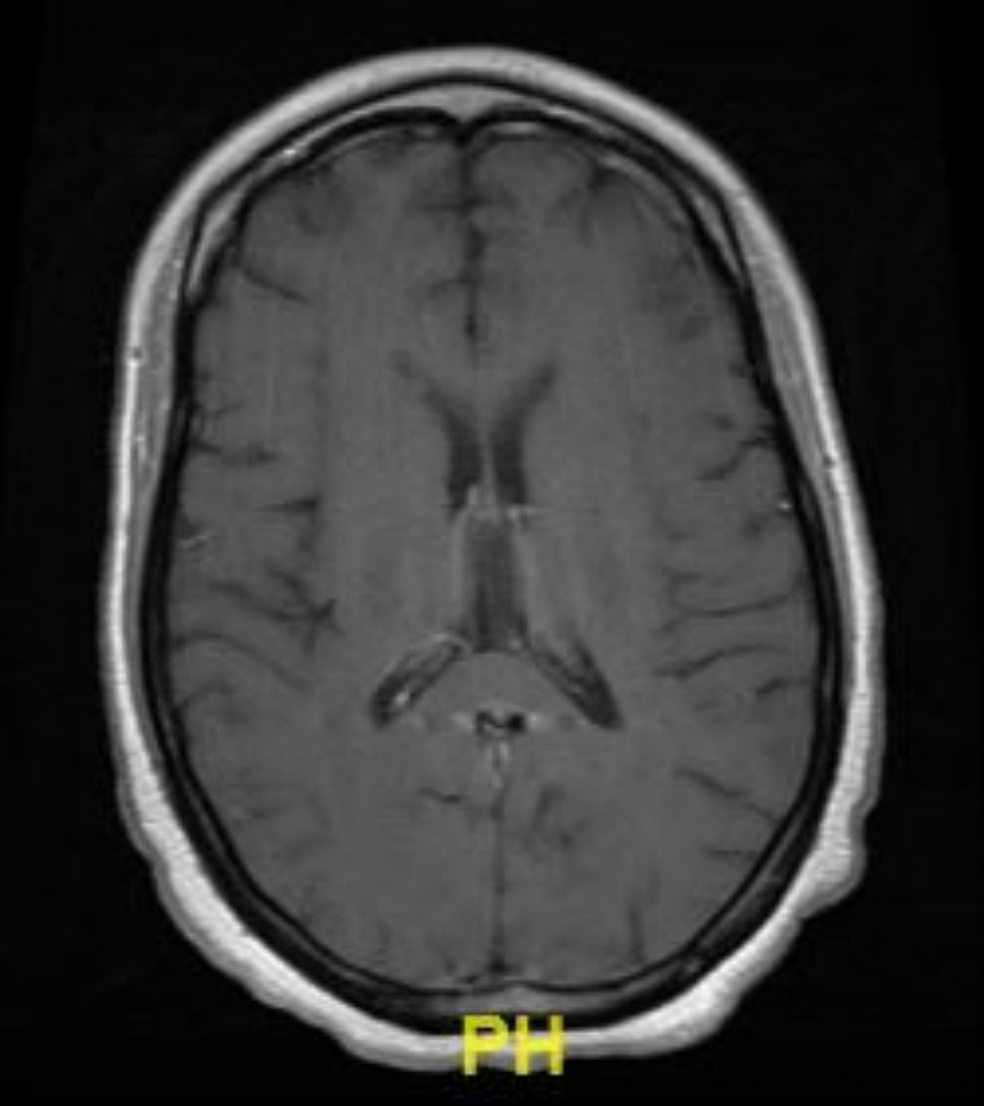
Magnetic resonance imaging with and without contrast with no acute infarction, hemorrhage, or mass.

At admission, complete blood cell count (CBC) and comprehensive metabolic panel (CMP) were unremarkable. Chest X-ray was unremarkable. Computed tomography angiography (CTA) and magnetic resonance imaging (MRI) of the head were negative for acute findings (Figure 1).

Lumbar puncture was performed with no acute findings. Cerebral spinal fluid (CSF) was clear and colorless in appearance with WBC 22/mm^3^, RBC 12/mm^3^, glucose 47 mg/dL, protein 43 mg/dL, and negative for xanthochromia. Syphilis serologies, HIV screen, and CSF herpes I/II polymerase chain reaction (PCR) were negative. Blood cultures showed no growth. There was no evidence of infection or clear infectious source. During hospitalization, the patient developed swelling of her fingers bilaterally, which prompted screening for rheumatological diseases. Antinuclear antibody (ANA), anti-Smith (anti-SM) antibody, double stranded DNA (dsDNA) were positive and she had erythrocyte sedimentation rate of 40 mm/hour. Complement C3 and C4 levels were within normal limits.

**Figure 2:**
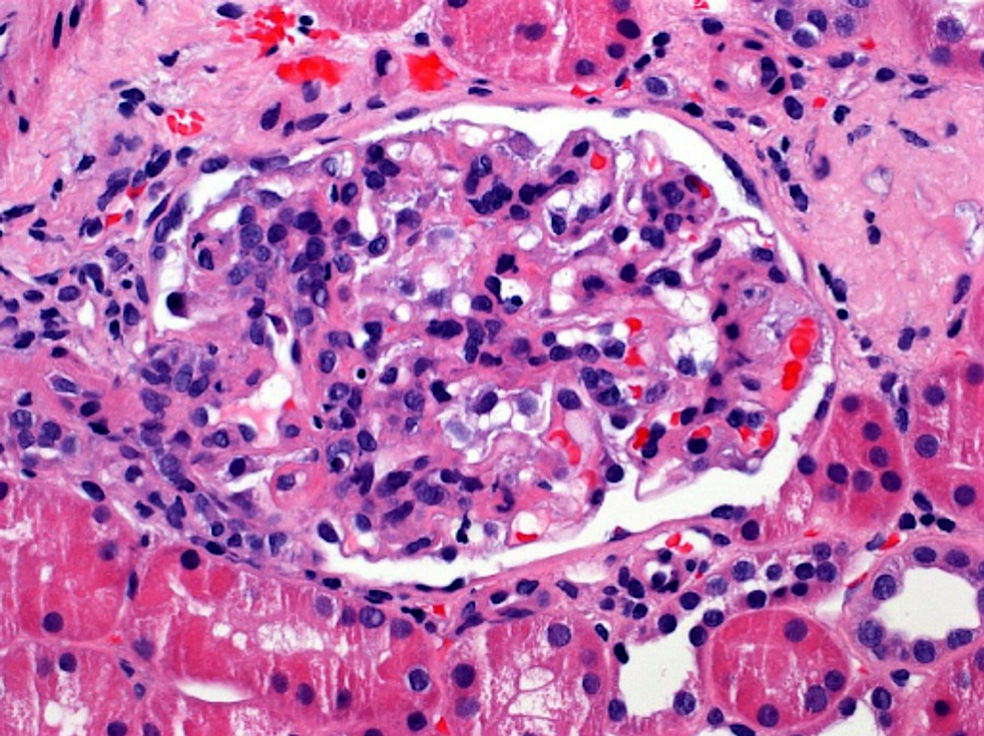
Biopsy positive for Focal Lupus Nephritis/Membranous Lupus Nephritis ISNRPS* Class V, mild activity seen with endocapillary proliferation. *International Society of Nephrology and the Renal Pathology Society

Urine studies showed proteinuria of 600 mg/dL and hematuria of 0.5 mg/dL warranting a renal biopsy which revealed Focal Lupus Nephritis, International Society of Nephrology and the Renal Pathology Society (ISNRPS) Class III, and Membranous Lupus Nephritis ISNRPS Class V (Figure 2).

The patient was subsequently diagnosed with SLE and started on prednisone, hydroxychloroquine and mycophenolate; however, psychosis persisted with this regimen. The patient was then started on quetiapine with no resolution in symptoms and was transitioned to olanzapine with improvement of her psychiatric symptoms.

## Discussion

SLE is an autoimmune disease with multiorgan involvement. The various psychiatric and neurologic manifestations that occur secondary to SLE involvement of the nervous system are collectively referred to as lupus cerebritis. These manifestations vary widely and can include headache, anxiety, depression, psychosis and pseudodementia. Psychosis occurs in about 5% of patients diagnosed with lupus and in our patient, this psychosis was characterized by visual hallucinations. These manifestations most often present within the first year of diagnosis [1]. Lupus cerebritis is a diagnosis of exclusion and the presentation of psychosis can be due to various etiologies that need to be evaluated; these include drug use, infections, structural brain abnormalities and metabolic abnormalities. Workup requires establishing a diagnosis of SLE through serologic markers. ANA will be positive in all patients with SLE but this marker is not specific, thus it is necessary also to test for markers such as Anti-Sm Ab and dsDNA. Low complement levels also help to reinforce the diagnosis of SLE; however they are unreliable as decrease in complement proteins can lead to increased synthesis resulting in complement levels remaining in normal range [2]. Renal biopsy can be critical in diagnosis of SLE as nearly half of all SLE cases involve the kidneys and is an important predictor of mortality [3]. A study by West et al. suggested that CSF findings may play a role in the diagnosis of lupus cerebritis with abnormal IgG, oligoclonal bands, antineuronal antibodies, and serum antiribosomal-P antibodies which are noted in complex cases [4]. Abnormal MRI findings including increased periventricular signaling and enlargement of the prepontine cistern may be noted particularly in those with focal neurologic deficits; however they are not specific for lupus cerebritis [5]. A study comparing MRI and positron emission tomography (PET) utility in 13 patients with active SLE with neuropsychiatric symptoms showed that only 15% of these patients had abnormal MRI findings, while 100% demonstrated hypometabolism abnormalities on PET scan [6]. However, it should be noted that these PET findings are not specific to lupus cerebritis. Electroencephalography (EEG) can also be considered as a tool for diagnosis of lupus cerebritis, with most abnormal EEGs in lupus cerebritis patient’s showcasing diffuse slow wave activity [7]. However, these EEG findings have both a low sensitivity and specificity for diagnosis.

For mild or moderate cases, azathioprine and mycophenolate are used with transition to high-dose glucocorticoids and intravenous cyclophosphamide in severe cases. If there is still no resolution in symptoms, rituximab, intravenous immunoglobulins, or plasmapheresis can be used [8]. It should be noted, however, that steroids can exacerbate psychosis, as seen in our patient. Often lower glucocorticoid dosages, addition of antipsychotics, or pulse dose cyclophosphamide are required for improvement of lupus-related psychosis [9].

## Conclusions

We present this case to increase awareness of the psychiatric manifestations of SLE that can be mistaken for more common etiologies of psychosis. In patients presenting with acute renal failure accompanied by AMS, it is important to consider psychiatric and neurologic manifestations of lupus nephritis. Initial steps in diagnosis of lupus cerebritis include diagnosing with SLE through biopsy and serological markers. It is also necessary to conduct a thorough workup for other causes of AMS. If workup for other etiologies of psychosis is negative and the clinician is able to confirm diagnosis of SLE, the clinician should consider initiating treatment for lupus cerebritis.
